# The manipulation of top-down interpretation as one’s symptomatic body reduces the sense of body ownership

**DOI:** 10.3389/fpsyg.2024.1399218

**Published:** 2024-12-23

**Authors:** Kazuki Yamamoto, Takashi Nakao

**Affiliations:** ^1^Graduate School of Humanities and Social Sciences, Hiroshima University, Higashihiroshima, Hiroshima, Japan; ^2^Japan Society for the Promotion of Science, Tokyo, Japan

**Keywords:** sense of body ownership, full-body illusion, top-down interpretation, depersonalization, skin conductance response

## Abstract

Sense of body ownership has been studied using rubber hand illusion (RHI) and full-body illusion (FBI). It has recently become clear that consciously interpreting a fake body as one’s own in a top-down manner influences these body illusions. Furthermore, a study interestingly found that the influence of top-down interpretation was moderated by the degree of depersonalization, which was a symptom of a lack of sense of body ownership. In a case study on depersonalization, the top-down interpretation of one’s body was suggested to be a negative physical state that made it difficult to feel a sense of body ownership. However, this has not been examined. We examined the influence of negative top-down interpretation using an FBI procedure. A fake body was instructed to be viewed as a negative self-body (“view the virtual body’s back while regarding the virtual body as your own experiencing abdominal pain”). To examine the influence of a negative top-down interpretation, participants were instructed to interpret the body as their own (neutral self-body) as a control condition. We used skin conductance responses to a fearful stimulus presented after an illusion procedure to measure the degree of FBI experienced. Results indicated a significant difference in the skin conductance response between the synchronous and asynchronous presentation of visual-tactile stimuli in the control condition, which confirmed the occurrence of the illusion. However, the occurrence of the illusion was not confirmed when the participants were instructed to interpret the virtual body as their own in a negative physical state, and the degree of FBI was smaller than the control condition. Our finding that an FBI was inhibited by manipulation of the top-down interpretation suggested that it could be a factor that inhibited the creation of a sense of body ownership.

## 1 Introduction

The feeling that “this body belongs to me” is called sense of body ownership (Gallagher, 2000). Philosophers and psychologists have discussed how this occurs for centuries. Previous studies have examined how a sense of body ownership is induced or inhibited (Costantini, 2014; Kilteni et al., 2015; Mottelson et al., 2023) and how it is plasticly regulated (de Jong et al., 2017; Roel Lesur et al., 2018) through body illusion phenomena, such as the rubber hand illusion (RHI; Botvinick and Cohen, 1998) and full-body illusion (FBI; Lenggenhager et al., 2007; Petkova and Ehrsson, 2008). In body illusion, individuals experience a sense of body ownership toward an object that is not their own body, such as a rubber hand or virtual body (Botvinick and Cohen, 1998; Lenggenhager et al., 2007; Petkova and Ehrsson, 2008). In the RHI, a visible rubber hand is stroked while the participant’s own hand is invisible. When one’s own hand and the rubber hand are stroked in spatiotemporal synchrony, one gradually feels that the rubber hand is their own (Botvinick and Cohen, 1998). Similarly, in the FBI, when a virtual body, presented in front of the participant [e.g., in a Virtual Reality (VR) environment], is stroked while their own back is also stroked, they gradually feel that the virtual body in front of them is their own body (Lenggenhager et al., 2007; Nakul et al., 2020). These illusions indicate that bottom-up factors, such as sensory processing that begins with external stimuli such as the spatiotemporal integration of visual-tactile information and proceed toward higher-level cognitive processes, are essential for the induction of a sense of body ownership.

Additionally, previous studies have demonstrated that conscious top-down interpretation, based on prior knowledge, expectations, and beliefs of the object, also influences and inhibits sense of body ownership. Beginning with Tsakiris and Haggard (2005) and further supported by several studies (Tsakiris et al., 2010; Guterstam et al., 2013), research has revealed that an illusion is less likely to occur when the objects used, such as wooden sticks, do not resemble the shape of a typical hand. This indicates that the cognitive interpretation that a wooden stick is not one’s own hand is part of the constraints that must be satisfied for external objects to be incorporated. However, the observed inhibition of the illusion in these studies may not solely result from top-down interpretative influences; it could also be affected by bottom-up factors. Specifically, the spatial alignment between the wooden stick and one’s own hand poses a challenge, suggesting that such manipulations might disrupt the synchronization of visual-tactile stimulation. Other studies have also used objects close to the self-body. Haans et al. (2008) found that a decrease in RHI strength when the texture of hand-shaped objects did not resemble human skin. Furthermore, in first-person FBI, visual information alone could produce the illusion (Carey et al., 2019). Although these results are discussed from a top-down interpretation, these studies did not explicitly manipulate participants’ top-down interpretations.

Recent studies have addressed concerns and demonstrated the influence of top-down interpretation by explicitly manipulating it while not interfering with a bottom-up input. Yamamoto and Nakao (2022) introduced a top-down interpretation with instruction (i.e., having the virtual body interpreted by instruction as their own or other’s bodies) that did not interfere with the bottom-up input. They found that the degree of FBI when the participants interpreted the virtual body as a self-body was higher than that when the participants interpreted the virtual body as another’s body, which indicated the influence of top-down interpretations on the illusion. Additionally, Berger et al. (2023) discovered that synchronous motor imagery of robot hand movements enhanced sense of body ownership and agency toward the robot hand when compared with asynchronous imagery. These studies demonstrate that top-down factors, such as interpretation of the virtual body and motor imagery, can also influence sense of body ownership.

Furthermore, Yamamoto and Nakao (2022) found that the influence of top-down interpretation was modulated by the degree of depersonalization, with a lack of sense of body ownership as the main symptom. They found that the higher the depersonalization tendency, the lower the degree of FBI when the virtual body was interpreted as their body. This result was interpreted based on hypotheses regarding the chronic process of depersonalization proposed in a review of case studies (Hunter et al., 2003). Hunter et al. (2003) stated that individuals who experienced depersonalization had false beliefs concerning their transient symptoms of depersonalization (e.g., permanent brain damage and impending death), which could lead to chronic depersonalization symptoms that were persistent and recurrent and significantly impaired daily life. Yamamoto and Nakao (2022) interpreted that individuals with depersonalization viewed their symptomatic bodies as targets to be evaluated negatively. Furthermore, when they consciously viewed a virtual body as their own, they negatively evaluated even the virtual body as their own, which could inhibit the occurrence of FBI. However, while they instructed participants to interpret the virtual body as a self-body, the instruction did not include a negative evaluation factor. Examining the possibility that top-down negative interpretations may inhibit the induction of body ownership is important for clarifying the contribution of top-down factors in the induction and inhibition mechanisms of sense of body ownership. This may also provide valuable insights, particularly for interventions aimed at patients with depersonalization who find it difficult to feel a sense of ownership over their own bodies.

This study aimed to clarify whether the top-down interpretation of a virtual body as one’s own, which exhibited a negative physical state, inhibited the FBI. To observe a relationship consistent with previous research (Yamamoto and Nakao, 2022) and examine the relationship with depersonalization, the experience of observing oneself from the outside (Sierra, 2009), we followed the experimental procedures described in Yamamoto and Nakao’s (2022) study. Specifically, we utilized the third-person perspective FBI paradigm (Lenggenhager et al., 2007) via VR, self-association instruction to a virtual body as a manipulation of top-down interpretation of the virtual body, and measurement of illusion via a questionnaire and skin conductance responses. The main difference was that we included a negative self-association condition, which instructed participants to interpret the virtual body as their own in a negative state (i.e., their own body experiencing abdominal pain). In this study, abdominal pain was used as a negative physical symptom. This choice was made because abdominal pain was a physical symptom that healthy individuals could experience. In addition, it was relatively easy for one to imagine their body in such a state. Given that physical pain was unpleasant and evoked negative emotions (Bresin et al., 2018), abdominal pain was considered an appropriate negative physical state. Based on the studies of Yamamoto and Nakao (2022) and Berger et al.’s (2023), which demonstrated the role of mental imagery in the modulation of bodily illusions, we explored how imagining the body in a state of abdominal pain affected the strength of the FBI.

The degree of illusion produced in the negative self-association condition was expected to be smaller than that produced in the neutral self-association condition, in which no specific instruction concerning a virtual physical state was provided, similar to in Yamamoto and Nakao’s (2022) study. This prediction was based on Hunter et al.’s (2003) notion that individuals with depersonalization were less likely to experience a sense of body ownership because they interpreted their physically symptomatic self-body as targets to be evaluated negatively.

Additionally, this study examined whether the negative correlation between the degree of depersonalization tendency (degree of depersonalization experiences among healthy individuals) and degree of FBI in the neutral self-association condition observed in Yamamoto and Nakao’s (2022) study could be replicated. Furthermore, we expected that this negative correlation would be difficult to observe when a negative interpretation was added. Therefore, we expected that the negative correlation observed in the neutral self-association condition would not be observed in the negative self-association condition. This expectation was based on the possibility that FBI suppression owing to negative interpretations of virtual bodies could occur for all the participants, regardless of depersonalization tendency, which thus diminished the correlation.

## 2 Materials and methods

### 2.1 Participants

In this study’s experiment, the experimenter (first author: KY) had to touch the participants’ backs during the FBI. Therefore, only male participants, the same gender as the experimenter, were recruited. Previous studies reported no significant differences in the degree of illusion between men and women (Petkova and Ehrsson, 2008; Kilteni et al., 2013). All participants underwent the experimental procedures through the same experimenter.

A total of 32 men participated in this study, of which 27 (mean age: 21.9 years, range: 18–26 years) were included in the analysis. We excluded five participants owing to failing to record their Skin Conductance Response (SCR), used as an index for the generation of illusion (criteria are described in detail in the analysis).

We did not estimate the sample size in prior. The sample size was determined via the results of a previous study (Yamamoto and Nakao, 2022) that compared the degree of FBI through top-down manipulations (top-down association with a virtual body through instruction). The study included 31 participants, with 27 used for analysis. The approach of basing the sample size on previous studies was practical when other information sources were lacking or when new manipulations were used (Althubaiti, 2022; Lakens, 2022). Our study’s main objective involved participants’ interpretation of a virtual body as their own in a negative state, which was a novel aspect. Owing to this unique aspect, we could not directly refer to effect sizes from previous studies. Therefore, we determined our sample size based on studies that conducted and compared similar manipulations.

This study was reviewed and approved by the Ethics Committee of the Graduate School of Human and Social Sciences, Hiroshima University (approval number: HR-PSY-000838). All participants provided written informed consent and were informed that they could discontinue their participation anytime. Upon completion of the experiment, the participants were compensated with 1,500 JPY.

### 2.2 Depersonalization questionnaire

This study utilized the Japanese version of the Cambridge Depersonalization Scale (CDS; Tanabe, 2004). The CDS comprised 29 items reported as depersonalization symptoms. These symptoms were a sub-classification of dissociative disorders in the Diagnostic and Statistical Manual of Mental Disorders Fourth Edition (DSM-IV) [DSM-V (American Psychiatric Association, 2013) has the same classification]. Each item comprised two Likert scales for depersonalization experiences in the last 6 months, with a 5-point and 6-point scale for frequency (0: never to 4: always) and duration (1: few seconds to 6: more than a week), respectively. Higher total scores indicated a more substantial likelihood of depersonalization. However, if the participant answered “0” for frequency, there would also be no duration. Consequently, this study added one point (0: “never, and therefore cannot be answered”) to the duration. The CDS score was calculated as the sum of these items, with a maximum and minimum score of 290 and 0, respectively. According to Sierra and Berrios (2000), a cut-off point of 70 was considered sensitive for patients with depersonalization. An example of the items include “Out of the blue, I feel strange, as if I were not real or as if I were cut off from the world.” Since the CDS was reportedly an effective screening tool for healthy participants (Sugiura et al., 2009), the term depersonalization tendency was treated as the degree of depersonalization experience in healthy individuals in this study. Furthermore, the CDS score was an indicator of depersonalization tendency in healthy individuals. Participants’ mean CDS score was 40.59 (*SD* = 20.37) and ranged from 7 to 102.

### 2.3 Equipment

This study used a head-mounted display (HMD) and an Oculus Rift (Oculus; display resolution: 1200 × 698) to immerse the participants in a VR environment. A ProLite G2773HS (iiyama) display was used for the VR environment.

A galvanic skin response (GSR) module and temperature sensor (Brain Products Inc.) were used to measure the electrical response of the participants’ skin and skin temperature, respectively. An eight-channel bipolar amplifier system (Brain Product Inc.) was used to amplify the electrical signals for each physiological index.

### 2.4 Stimulation

To induce the illusion, a visual stimulus (an animation of stroking a hand up and down 15 cm on the back of the virtual body for 90 s) and tactile stimulus (stroking 15 cm of the participant’s back with a stick for 90 s) were used. Occurrence of the illusion was reportedly stable at 87 s (Finotti et al., 2023). Therefore, in this study, the stimulus presentation time was set to 90 s.

To elicit the SCR used to measure the degree of illusion, a fear stimulus (animation of a knife in the back of the virtual body) was presented after the visual-tactile stimuli were presented for 90 s.

**Figure 1:**
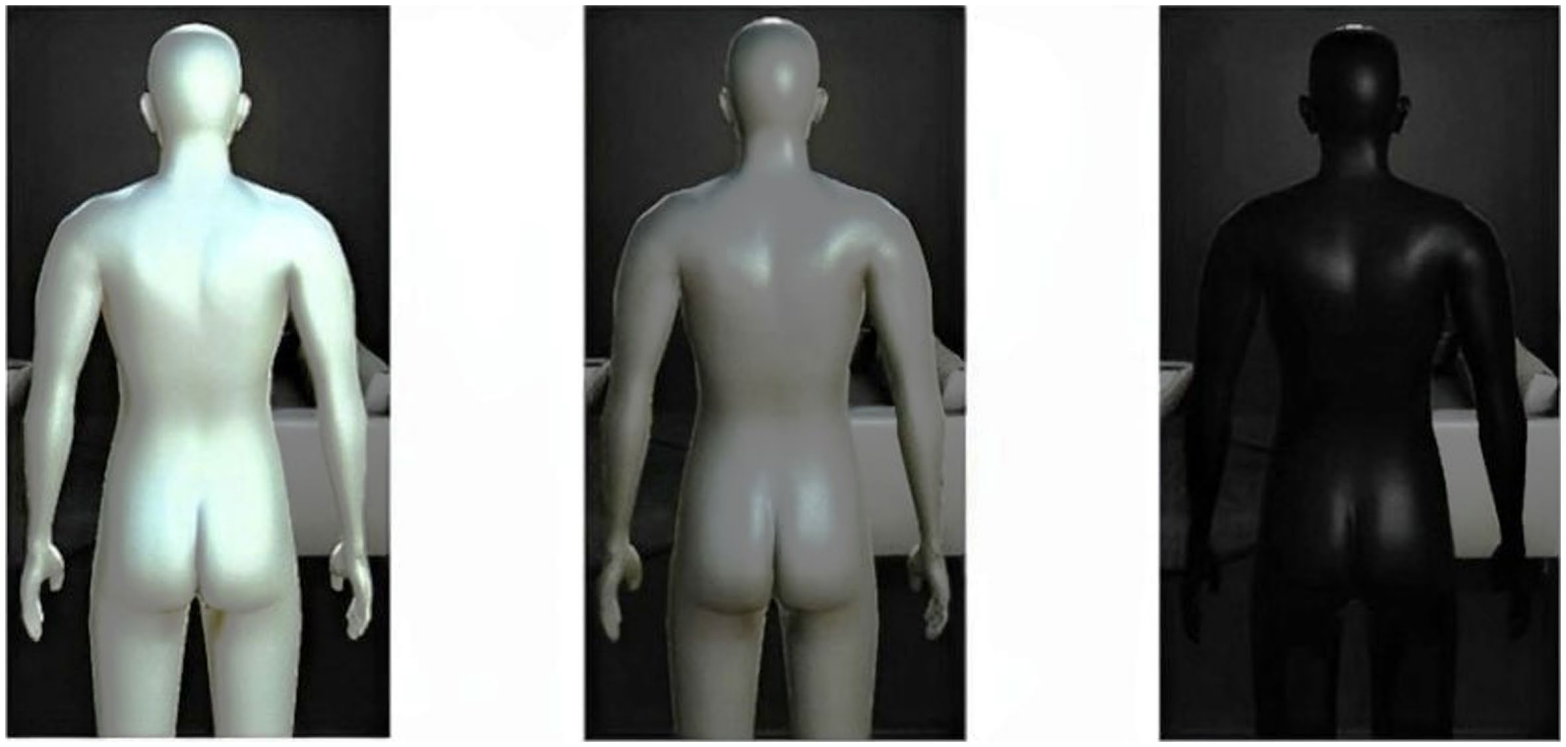
The three virtual bodies used in this study. Colors were from left to right: white (R: 255, G: 255, B: 255), gray (R: 127, G: 127, B: 127), and black (R: 0, G: 0, B: 0).

The lightness of the skin color of the virtual body used in each association condition was changed (Figure 1) to avoid confusion in top-down interpretation between the conditions. For example, if we used a white avatar in the negative self-association condition and subsequently presented the same white avatar in the neutral self-association condition, a concern arose that the initial negative interpretation associated with the white avatar could make it difficult to newly interpret it as the neutral self-body.

### 2.5 Experimental design

This experiment included two within-participant factors. First, the presentation of visual-tactile stimuli, which included synchronous and asynchronous conditions. Second, top-down body association, which included three conditions: non-association, neutral self-association, and negative self-association. Synchronous and asynchronous conditions were included for each association condition. The participants participated in all experimental conditions.

Regarding visual-tactile stimuli, the synchronous condition induced illusions, whereas the asynchronous condition did not. In the synchronous condition, the visual and tactile stimulus, movement of the hand stroking the back of the virtual body in VR and stick stroking the participant’s back, respectively, were synchronized. In the asynchronous condition, when the hand stroking the back of the virtual body in VR moved from top to bottom, the stick stroking the participant’s back moved from bottom to top. Conversely, when the hand stroked from bottom to top in the VR, the stick stroked the participant’s back from top to bottom.

Regarding the top-down body association instruction, a self-association condition, in which the virtual body was associated with the self (i.e., “view the virtual body’s back while regarding the virtual body as your own”) and negative self-association condition, in which the virtual body was associated with the self in a negative physical state (i.e., “view the virtual body’s back while regarding the virtual body as your own experiencing abdominal pain”), were introduced. No specific instructions were provided regarding the intensity or perspective of the imagined pain. This could include somatosensory imagery from the first-person perspective or visual imagery from the third-person perspective.

Additionally, to replicate the negative correlation between depersonalization tendency and degree of FBI in the neutral self-association condition (Yamamoto and Nakao, 2022), this study established a non-association condition [a typical full-body illusion procedure (Lenggenhager et al., 2007)]. Previous studies on depersonalization suggested that individual differences in SCR could reflect individual differences in the degree of depersonalization tendency independent of illusion creation (Michal et al., 2013; Horn et al., 2020). Yamamoto and Nakao (2022) calculated the mean SCR of the initially conducted non-association condition as the individual SCR independent of the FBI. They used it as a control variable when they examined the relationship between depersonalization tendency and degree of FBI. Consequently, to replicate these relationships, we also used the mean SCR in the non-association condition as the control variable.

### 2.6 Indices

The SCR and illusion questionnaire were used. However, given that this study’s instruction (e.g., “view the virtual body’s back while regarding the virtual body as your own”) may have encouraged the demand characteristics in the illusion questionnaire, SCR was predominantly used as an index of creation of the illusion (Petkova and Ehrsson, 2008; Guterstam et al., 2015). However, considering that sense of body ownership was a subjective experience (Gallagher, 2000), the illusion questionnaire, which could identify the subjective aspect of illusion creation, was not excluded from the analysis. Rather, it was treated as a secondary illusion index.

### 2.7 Skin conductance response

Notably, SCR was analyzed when a fear stimulus (animation of a knife in the back of the virtual body) was presented after visual-tactile stimuli were presented. The larger the FBI, the larger the SCR to the fear stimulus.

### 2.8 Illusion questionnaire

The illusion questionnaire inquired about the participants’ experience of the illusion during the visual-tactile stimuli presentation for 90 s. Questionnaire items were selected from the full-body illusion questionnaire used by Petkova and Ehrsson (2008) and Romano et al. (2014) and were based on the generated experiences during the third-person FBI. The illusion questionnaire consisted of five illusion items to measure the degree of FBI and three control items to determine whether the participants complied with the FBI task (Table 1). Participants responded to each item on a 7-point Likert scale (range: −3–3; label: −3, “not applicable,” 0, “neither agree nor disagree,” and 3, “frequently applicable”).

**Table 1:** Items of the illusion questionnaire.

Q1 (Illusion item): It felt like the virtual body was my own body
Q2 (Control item): I felt naked
Q3 (Control item): It felt as if my own body had turned into a virtual body
Q4 (Control item): I felt as if I had two bodies
Q5 (Illusion item): It seemed as though the touch I felt was caused by the hand touching the virtual body (= I felt tactile sensations from the virtual body)
Q6 (Illusion item): I felt as if I was drifting frontwards or backwards
Q7 (Illusion item): It felt like I could control the movement of the virtual body I was looking at
Q8 (Illusion item): I felt like I could not move my own body

### 2.9 Procedure

Given that this study measured SCR, the room temperature was kept constant for all the participants. The room’s heating was turned on and adjusted 1 h before the participants were scheduled to visit to ensure a room temperature of 24°C.

The experiment was explained to the participants after they entered. At that time, the participants were not informed of the FBI; however, they were informed that the study involved a psychological experiment in a VR environment. Subsequently, the participation consent form was explained. After consent to participate was obtained, the electrodes to measure the SCR were attached to the upper first joints of the index and middle fingers of the participant’s left hand.

Participants were asked to wear the HMD after being told how to adjust its size and focus. To eliminate differences between the participant’s viewpoint in the VR environment and real participant’s viewpoint, the height of the participant’s viewpoint in the VR environment was automatically adjusted via the Oculus Sensors to detect the height of the HMD in the real environment. Subsequently, participants were asked to adjust their body orientation and position to ensure that their viewpoint was directly facing the back of the virtual body, which allowed them to view the entire back.

Individuals who were unfamiliar with VR environments could experience sickness during immersion. Therefore, to minimize the possibility of VR sickness, participants were asked to wear an HMD before the experiment and immersed in the actual experimental environment for 3 min. During this time, participants were asked to move their heads freely to familiarize themselves with the VR environment. After cues were received from the participants, which indicated that they were familiar with the VR environment, they proceeded to the FBI experimental phase.

Regarding the FBI experimental phase, a non-association condition was always performed first for all the participants. This was done to avoid the effects of instructions being carried over to the non-association condition by conducting neutral and negative self-association conditions before the non-association condition.

Regarding the non-association condition, participants were asked to wear the HMD at the beginning of the trial, and the synchronous and asynchronous conditions were conducted twice. The order of the synchronous and asynchronous conditions was randomized for each participant. While the visual-tactile stimuli were presented for 90 s, participants were instructed to stand and look at the virtual body’s back. After 90 s of stimulus presentation, a fear stimulus was presented (animation of a knife in the back of the virtual body). After the presentation of the fear stimulus, participants were asked to answer the illusion questionnaire. After they answered the illusion questionnaire, participants proceeded to the subsequent trial.

In the neutral or negative self-association condition, participants were instructed to associate the virtual body with their own or negative self-body (e.g., “view the virtual body’s back while regarding the virtual body as your own” or “view the virtual body’s back while regarding the virtual body as your own experiencing abdominal pain”) before the presentation of the visual-tactile stimuli. After the instructions, participants were asked “Let me (the experimenter) know when you are able to regard that way.” After the experimenter received a signal from the participant, the visual-tactile stimulus was presented. The order in which the synchronous and asynchronous conditions were conducted after the instructions was randomized for each participant. All participants, in every condition, received tactile stimuli from the same experimenter (first author: KY), who had been thoroughly trained to synchronize stroking the participant’s back with the visual stimuli prior to the experiment.

After all the association conditions were completed, the electrode attached to the participant’s left hand was removed. Finally, the participants were asked to complete a gratuity transfer form.

### 2.10 Statistics

#### 2.10.1 Skin conductance response

Skin conductance responses were measured for 5 s after the presentation of the fear stimulus. Recorded skin electrical activity was analyzed using Ledalab 3.2.5 (Benedek and Kaernbach, 2010) on MATLAB 9.7.0 (MathWorks). Continuous Decomposition Analysis (CDA) was used to separate the skin conductance level (SCL), which entailed a gradual change in current conductance, from the skin conductance response (SCR), which entailed a transient response. This method effectively minimized the influence of SCL activity on SCR measurements. The SCR (CDA.SCR) was measured within 5 s of stimulus presentation and calculated as the mean value for each participant for the synchronous and asynchronous cutaneous electric response conditions. In this study, based on Petkova and Ehrsson’s (2008) study, participants with a CDA.SCR of 0 in more than two-thirds of the trials were considered to have experienced a problem in recording the skin electrical response; consequently, five participants were excluded from the analysis.

#### 2.10.2 Illusion questionnaire

An illusion questionnaire was administered to each participant by averaging the illusion and control items in each stimulus presentation for each association condition.

#### 2.10.3 Statistical analyses

We utilized R (version 4.3.2) and R Studio (version 2023.12.1 + 40) for statistical analysis. Prior to comparison of the mean differences, a Shapiro–Wilk test was performed to assess normal distribution of the data. A non-parametric test was applied for any combination of data that did not meet the normal distribution criteria (Shapiro–Wilk <0.05). This method maintained the consistency, robustness, and clarity of our analyses.

For the primary comparisons, all analyses were planned *a priori*. Paired two-group difference tests were used for all the comparisons. Each comparison was based on independent hypotheses, so multiple comparison corrections were not used. When the Wilcoxon signed-rank test was conducted, a continuity correction was applied. Furthermore, the effect size was calculated by dividing the *Z*-value by the square root of the sample size. The bootstrap method was used to calculate 95% confidence intervals, with the number of samples set to 1,000 (set seed = 1,234).

For primary correlations, all analyses were planned a priori. Correlations were conducted via data from two variables. Each correlation was based on independent hypotheses, so no corrections for multiple comparisons were made. A Shapiro–Wilk test was first used to check for normal distribution in the data when examining the correlation between the two datasets. A non-parametric test was applied to combinations that did not follow normal distribution (Shapiro–Wilk <0.05). Spearman’s rank correlation analysis utilized the bootstrap method to calculate 95% confidence intervals for the effect size (sample size = 1,000; set seed = 1,234).

## 3 Results

### 3.1 Confirmation of full-body illusion creation

**Figure 2:**
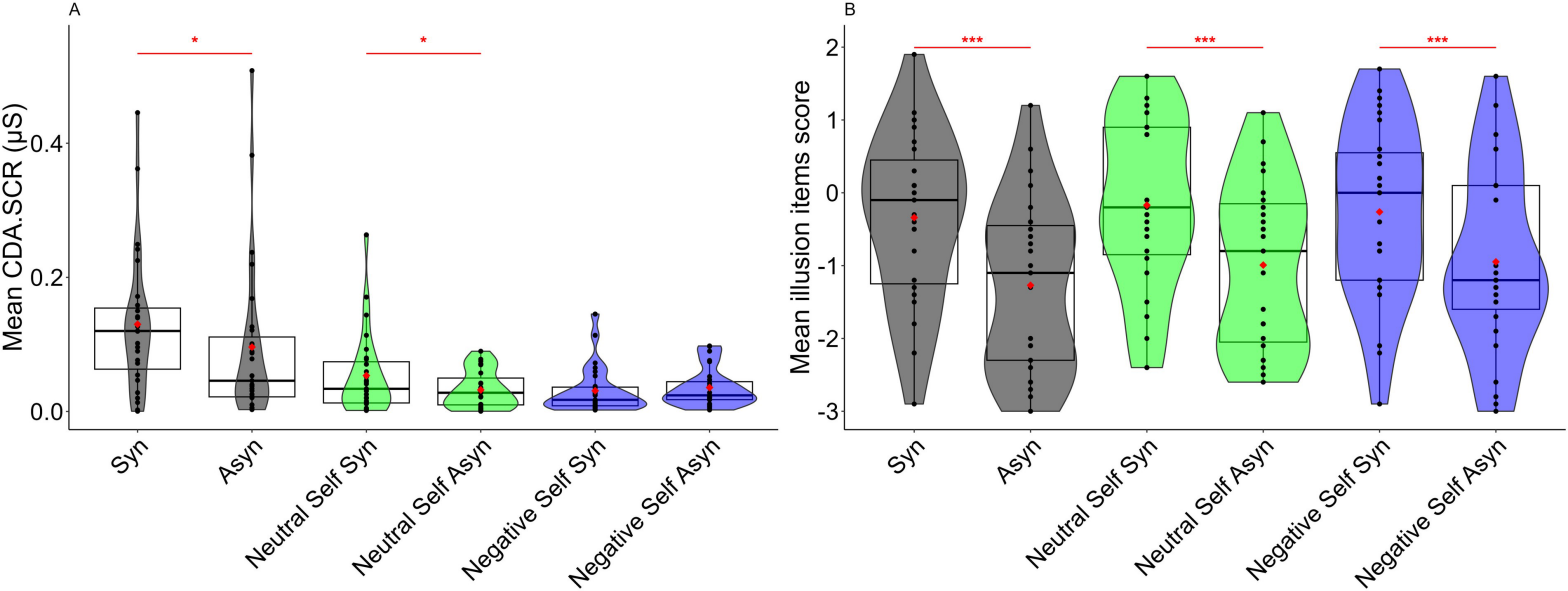
**(A)** Mean CDA.SCR for the synchronous and asynchronous conditions for each top-down body association condition. **(B)** Mean illusion items score for the synchronous and asynchronous conditions for each top-down body association condition. Box-and-whisker plot displays the median, lower/higher quantile, and minimum/maximum of the individual data. Red diamonds indicate the average values (***p < 0.001, *p < 0.05). See Supplementary material for descriptive statistics for each condition.

First, to confirm the creation of the FBI, the synchronous and asynchronous conditions were compared for each association condition (non-association, neutral self, and negative self). Regarding the SCR, the Wilcoxon’s signed rank test indicated a significant difference between the synchronous and asynchronous condition in both the non-association and neutral self-association conditions, with higher CDA.SCR in the synchronous condition (non-association condition: *Z* = 2.439, *p* = 0.015, *r* = 0.469, 95% CI = 0.095, 0.742; neutral self-association condition: *Z* = 2.126, *p* = 0.033, *r* = 0.409, 95% CI = 0.049, 0.701; Figure 2A). However, no significant differences were observed in the negative self-association condition (*Z* = −0.877, *p* = 0.381, *r* = 0.169, 95% CI = 0.002, 0.535; Figure 2A).

Regarding the illusion questionnaire, similarly, the Wilcoxon’s signed-rank test of the illusion item scores showed significant differences in all the association conditions, with significantly higher scores in the synchronous condition than in the asynchronous condition (non-association condition: *Z* = 3.536, *p* < 0.001, *r* = 0.680, 95% CI = 0.429, 0.857; neutral self-association condition: *Z* = 4.162, *p* < 0.001, *r* = 0.801, 95% CI = 0.683, 0.860; negative self-association condition: *Z* = 3.641, *p* < 0.001, *r* = 0.701, 95% CI = 0.456, 0.850; Figure 2B).

In difference to the SCR, the illusion questionnaire also showed significant differences in the negative self-association condition. This discrepancy could be due to the possibility that the instruction addressed in this study distorted the responses to the illusion questionnaire. Specifically, the inclusion of “view the virtual body’s back while regarding the virtual body as your own” in the instructing may have moved the value in a positive direction even in the synchronous condition of the negative self-association condition, which made it difficult to create the illusion. Since the influence of demand characteristics were unlikely to enter into the skin conductance response, we concluded that illusions occurred in the non-association and neutral self-association conditions.

### 3.2 Influence of negative top-down interpretation of the virtual body on full-body illusion

**Figure 3:**
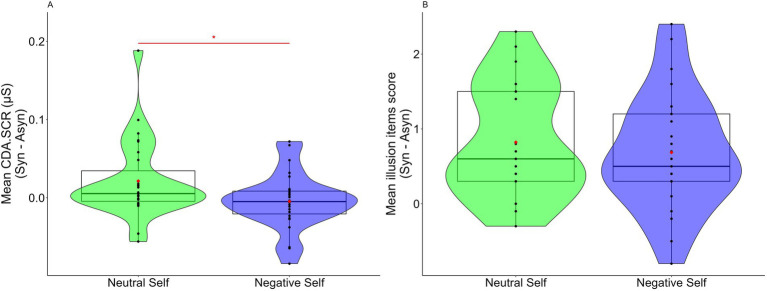
**(A)** Value of the difference in CDA.SCR between the synchronous and asynchronous conditions in the neutral and negative self-association conditions. **(B)** Value of the difference in the illusion items’ scores between the synchronous and asynchronous conditions in the neutral and negative self-association conditions. The box-and-whisker plot displays the median, lower/higher quantile, and minimum/maximum of the individual data. Red diamonds indicate the average values (*p < 0.05).

To examine whether the top-down interpretation of the virtual body as a self-body with negative physical symptoms influenced FBI, the degree of illusion (difference between synchronous and asynchronous conditions) in the neutral and negative self-association conditions were compared. Regarding SCR, a significant difference was observed between the two conditions, which indicated that CDA.SCR was higher in the neutral self-association condition than in the negative self-association condition (*Z* = 2.159, *p* = 0.031, *r* = 0.415, 95% CI = 0.073, 0.704; Figure 3A).

However, no significant difference was observed for the illusion questionnaire (*Z* = 0.978, *p* = 0.328, *r* = 0.188, 95% CI = 0.010, 0.528; Figure 3B).

### 3.3 Replication of the relationship between the degree of depersonalization tendency and the degree of illusion in the neutral self-association condition

**Figure 4:**
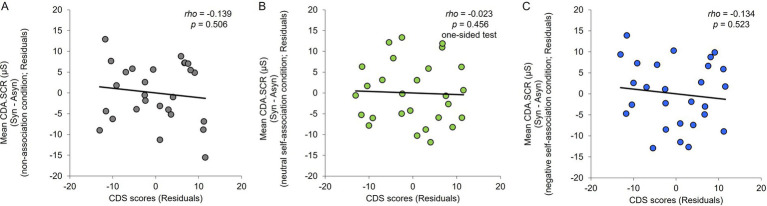
**(A)** Scatter plots for the CDS scores and value of the difference in CDA.SCR between the synchronous and asynchronous conditions in the non-association condition. **(B)** Scatter plots for the CDS scores and value of the difference in CDA.SCR between the synchronous and asynchronous conditions in the neutral self-association condition. **(C)** Scatter plots for the CDS scores and value of the difference in CDA.SCR between the synchronous and asynchronous conditions in the negative self-association condition. Control variables were age and overall mean of the non-association condition.

This study aimed to confirm whether the negative correlation between the degree of depersonalization tendency and degree of illusion (for the SCR) in the neutral self-association condition in Yamamoto and Nakao’s (2022) study was also observed in this study. Spearman’s rank correlation analysis was conducted between the CDS scores and degree of illusion (difference in the CDA.SCR between the synchronous and asynchronous conditions) for each association condition. Similar to Yamamoto and Nakao’s (2022) study, this study used the means of the CDA.SCR for the four trials in the non-association condition, and age as a control variable. Furthermore, given that a negative correlation was expected, the relationship between the degrees of depersonalization and illusion in the neutral self-association condition was one-tailed (Yamamoto and Nakao, 2022). The results indicated no significant correlation with the degree of depersonalization tendency in any of the association conditions (non-association condition: *rho* = −0.139, *p* = 0.506, 95% CI = −0.608, 0.377; neutral self-association condition: *rho* = −0.023, *p* = 0.456, 95% CI = −0.426, 0.330, one-sided test with higher boundary; negative self-association condition: *rho* = −0.134, *p* = 0.523, 95% CI = −0.539, 0.307; Figure 4).

**Figure 5:**
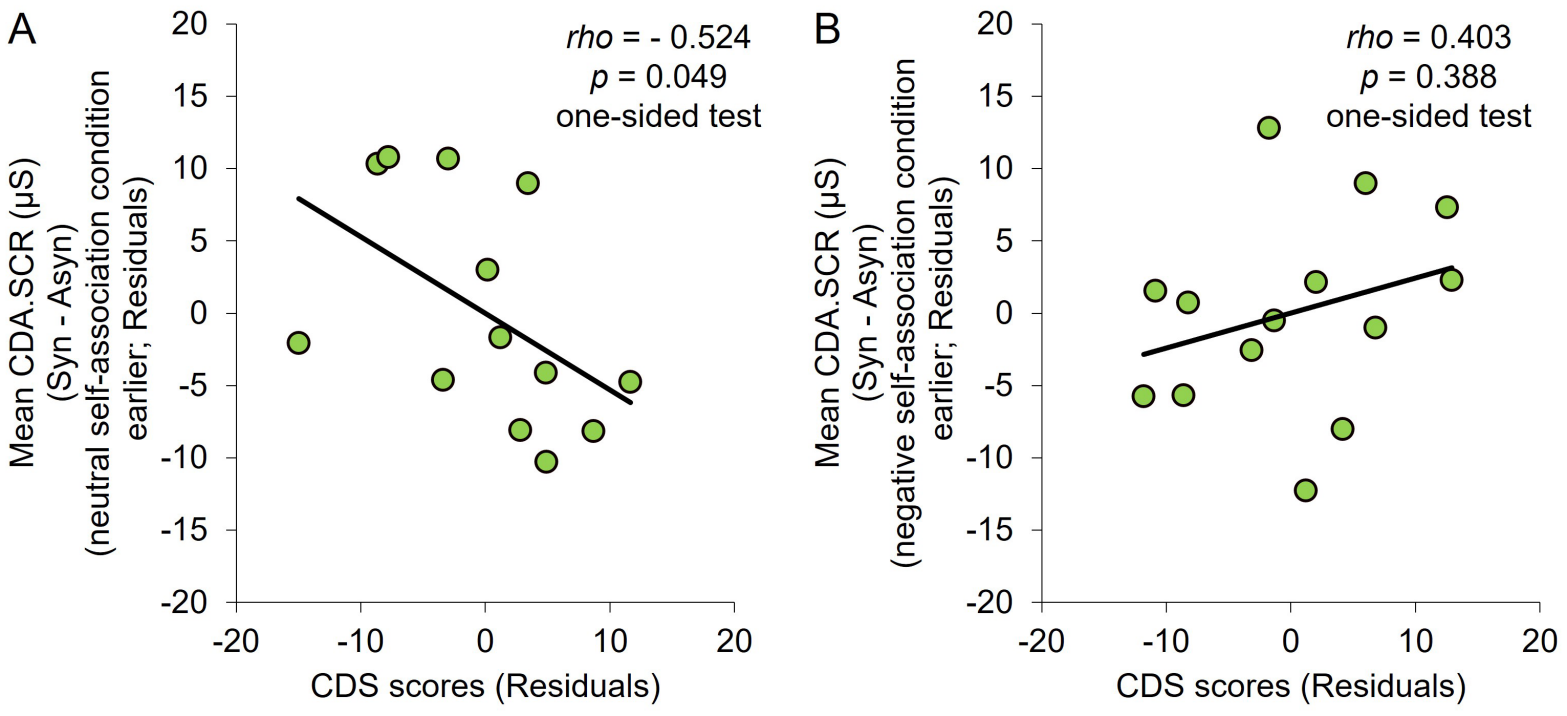
**(A)** Scatter plots for the CDS scores and value of the difference in CDA.SCR between the synchronous and asynchronous conditions in the neutral self-association condition for the group who conducted the neutral self-association condition earlier. **(B)** Scatter plots for the CDS scores and value of the difference in CDA.SCR between the synchronous and asynchronous conditions in the neutral self-association condition for the group who conducted the negative self-association condition earlier. Control variables were age and overall mean of the non-association condition.

This study found no negative correlation between the degree of depersonalization and degree of illusion in the neutral self-association condition, similar to Yamamoto and Nakao’s (2022) results. This may have been due to the influence of the negative top-down interpretation being carried over to the participants who participated in the neutral self-association condition after the negative self-association condition. Therefore, the correlation was analyzed by dividing the participants into two groups of those who conducted the neutral self-association condition earlier (13 participants) and conducted the negative self-association condition earlier (14 participants). The results indicated a significant negative correlation in the group who conducted the neutral self-association condition earlier (*rho* = −0.524, *p* = 0.049, 95% CI = −0.882, 0.190, one-sided test with higher boundary; Figure 5A). However, no significant correlation was observed in the group who conducted the negative self-association condition earlier (*rho* = 0.403, *p* = 0.388, 95% CI = −0.265, 0.841, one-sided test with higher boundary; Figure 5B).

## 4 Discussion

### 4.1 Influence of “manipulation” of the negative top-down interpretation of the virtual body on full-body illusion

This study aimed to clarify whether top-down interpretation of a virtual body as one’s own, which exhibited a negative physical state, inhibited FBI. This study adopted a procedure in which participants were instructed to interpret the virtual body as a self-body in a state of abdominal pain and as a self-body that exhibited negative physical symptoms. The results indicated that illusion inhibition was observed in the CDA.SCR (Figure 2A), and the degree of illusion was smaller than when the participants were instructed to interpret the virtual body top-down as a neutral self-body (Figure 3A). This result indicated that “manipulating” the top-down interpretation of a virtual body as the self in a negative physical state inhibited the FBI. There was a reason why we did not state that the negative top-down interpretation factor inhibited the FBI but referred to it as a “manipulation” of the negative top-down interpretation. This was because, apart from the possibility that a factor, such as negative interpretation could have inhibited the FBI, another factor could have been considered. This point has been discussed in further detail below.

Regarding the illusion questionnaire, no significant difference was observed between the neutral and negative self-association conditions (Figure 3B). A reason for this could have been that both instructions included “regard the virtual body as your own.” Given that the illusion questionnaire included the item “I felt like the virtual body was my body,” the demanding characteristic of responding per the instruction may have been reflected in both conditions, which resulted in no significant difference being observed. However, owing to the SCR that reflected the activity of the autonomic nervous system, it could be assumed that the effect of demand characteristics was less likely to be reflected in the SCR.

### 4.2 Why the full-body illusion was inhibited by the “manipulation” of negative top-down interpretation

We discuss two reasons why the FBI was inhibited by the manipulation of (not as the factor of) a negative top-down interpretation. First, the negative top-down interpretation of the self-body may have inhibited the FBI, as hypothesized in this study. The negative top-down interpretation of the self-body inhibited the FBI owing to the sense of depersonalization that accompanied the sense of body ownership of the self-body. Based on the argument that depersonalization was a defense mechanism against danger and associated anxiety (Ananthaswamy, 2015/2018), the reason negative top-down interpretation inhibited the FBI may be considered from a defense mechanism perspective. Therefore, when the virtual body was interpreted as a self-body in a state of abdominal pain, the FBI may be inhibited in an attempt to avoid a negative physical state.

Second, the difference in the physical state between the actual self-body and virtual body may have been influenced. Participants were instructed to interpret the virtual body as their own body with abdominal pain; however, their actual body was not in such a state. Therefore, they experienced difficulty interpreting the virtual body as their own body, which may have inhibited the FBI. Considering that the RHI and FBI were not created while visual-tactile stimuli were presented asynchronously (Botvinick and Cohen, 1998; Lenggenhager et al., 2007; Petkova and Ehrsson, 2008) and that the FBI was not created in the other-association condition (Yamamoto and Nakao, 2022), the FBI may have been inhibited when participants had difficulty interpreting the virtual body as their own body, regardless of bottom-up or top-down factors.

Therefore, this study observed that the “manipulation” of negative top-down interpretation prevented the FBI. Furthermore, whether the negative top-down interpretation or difference in the physical state between the self-body and the virtual body inhibits the creation of the FBI. Future studies should examine the factors inhibiting this illusion.

### 4.3 Relationship between depersonalization tendency and full-body illusion

This study also examined whether the negative correlation between depersonalization tendencies and degree of FBI in the neutral self-association condition, observed by Yamamoto and Nakao’s (2022) study, could be replicated. Regarding the neutral self-association condition, when participants were divided into two groups based on the order in which the neutral self-association condition was conducted, and the relationships were analyzed, a significant negative relationship was observed in the group that conducted the neutral self-association condition earlier (Figure 5A). However, no significant relationship was observed in the negative self-association condition groups (Figure 5B). This suggested that the incongruence between Yamamoto and Nakao’s (2022) study and this study’s replication was due to the influence of the negative self-association condition being carried over to the neutral self-association condition, even in the neutral self-association condition. Furthermore, no significant relationship was observed between the degree of depersonalization tendency and degree of the FBI in the negative self-association condition (Figure 4C).

The two above-mentioned possibilities could also explain the results of this study regarding the relationship between depersonalization tendencies and the FBI. First, a negative interpretation may inhibit the FBI. Individuals with depersonalization viewed their symptomatic bodies as targets to be evaluated negatively (Hunter et al., 2003). Hence, when individuals with depersonalization consciously viewed a virtual body as their own body, they negatively evaluated the virtual body, which might have resulted in a negative correlation in the neutral self-association condition. Regarding the negative self-association condition, the negative interpretation may have inhibited the FBI, regardless of the degree of depersonalization tendency; thus, no significant correlation was observed.

Second, the difference in physical state between the self-body and virtual body may have inhibited the FBI. The cognitive load may have increased when interpreting the virtual body as a self-body and attention to visual-tactile stimuli was attenuated. A trade-off existed between attention to internal thoughts and attention to external stimuli, such as vision and tactile sensations (Ananthaswamy, 2015/2018). A negative relationship between the degree of depersonalization tendency and degree of the FBI in the neutral self-association condition was observed in this study (Figure 5A). This suggested that in individuals with a high depersonalization tendency, neutral self-association instruction may have diverted attention to thought, which attenuated attention to visual-tactile stimuli and inhibited the FBI. Previous studies reported that depersonalized individuals tended to ruminate (Quigley et al., 2022). That attention to thoughts may attenuate attention to perception, which resulted in depersonalization, such as separation between the physical and conscious self (Ciaunica et al., 2022). The possibility that attention to thoughts inhibited the creation of the FBI also applied to the negative self-association condition. This study manipulated the virtual body to be interpreted as a self-body in a state of abdominal pain; however, the self-body did not have such a state. Therefore, this may have caused an overload in thinking to recall the actual body in a state of abdominal pain and attenuated attention to the visual-tactile stimuli.

The following two points should be noted regarding the interpretation of the relationship between depersonalization tendency and full-body illusion. First, the replication of Yamamoto and Nakao’s (2022) result obtained in this study was observed when grouping was used. Hence, it is important to consider that grouping reduced the sample size, and thus, the detection power. Therefore, it is necessary to remove the influence of negative interpretation when interpreting the virtual body as a neutral self-body and ensure an appropriate sample size before reexamining this issue in the future. Second, regarding the lack of significant correlation observed in the negative self-association condition, our original expectation was that the negative correlation observed in the neutral self-association condition would become less apparent with the addition of a negative interpretation manipulation. To demonstrate this, an examination of the differences between the two correlations is necessary. However, contrary to our expectations, no negative correlation was observed in the neutral self-association condition across all the participants. Furthermore, the negative correlation was only observed when dividing the participants by the order of condition implementation. This made it difficult to detect the difference in correlations owing to the small sample size. Therefore, although we focused on interpreting the results for the negative self-association condition (Figure 4C), future research should aim to include a larger sample size and possibly alternative methodologies to further investigate the relationship between depersonalization tendencies and occurrence of illusions.

### 4.4 Concerns that presentation of a knife stimuli and image of abdominal pain distorted the results

The knife stimulus may have been interpreted by participants as the virtual body being in a state that would cause pain, similar to the negative top-down manipulation that caused the participants to interpret the virtual body as a negative physical state. Considering that physical pain is uncomfortable and causes negative emotions (Bresin et al., 2018), this may have evoked a negative interpretation of the virtual body. Specifically, the sequence of presentation of the negative event, that is, fear stimulus to the virtual body immediately after the virtual body was experienced as the self-body due to the synchronization of visual-tactile stimuli, which may have formed a negative interpretation of the self-body. Simultaneously, the recognition that the knife was not stuck and perceived physical pain in the self-body may have made participants interpret the virtual body and self-body as being in different states.

Considering the above discussion, presentation of the fear stimulus may have evoked a top-down interpretation that inhibited the FBI. Therefore, in the synchronous condition in which the fear stimulus was presented, participants might have been less likely to feel the virtual body as their own body and less likely to generate SCR in the subsequent trials. However, the FBI was still observed in the neutral self-association condition (Figure 2A). Thus, there was no evidence that the fear stimuli triggered a top-down interpretation that inhibited the FBI. Furthermore, similar to this study, previous studies (Ehrsson et al., 2007; Guterstam et al., 2015) that presented a fear stimulus to an object that induced an illusion reported that the illusion was maintained in the subjective measure, even after the procedure to induce the illusion and presentation of the fear stimuli. This also suggested that the fear stimuli itself was unlikely to have elicited the top-down interpretation that inhibited the FBI. However, this study did not measure participants’ subjective reports of top-down interpretations of fear stimuli. Therefore, future studies should consider the possibility that the presentation of fearful stimuli may induce a top-down interpretation that inhibits illusions. Measurement of participants’ subjective reports of top-down interpretations would be desirable.

Additionally, considering that skin conductance response was reactive to aversive stimuli, the aversive imagery of abdominal pain could have increased SCL and affected SCR amplitude. Therefore, we examined whether there was a difference in SCL between the neutral self-association condition and negative self-association condition during the 5 s after threat stimulus presentation. For this analysis, we used the average SCL of four trials without considering synchronous and asynchronous conditions. The results showed no significant difference between the conditions (*Z* = 0.076, *p* = 0.939, *r* = 0.015, 95% CI = 0.005, 0.440). This result indicated that there was no evidence that emotional imagery elevated SCL in our study.

### 4.5 Integrative interpretation of the results with a predictive coding model

The findings of this study can be framed within the predictive coding model of multisensory body illusions, which has been used to interpret the multisensory illusion phenomena (Apps and Tsakiris, 2014; Zeller et al., 2014, 2016; Rossi Sebastiano et al., 2021, 2024). The predictive coding model suggests that the embodiment of a fake hand results from the top-down modulation of lower inter-sensory conflicts emerging during the illusion procedure.

We regard the possibility that negative top-down interpretation inhibited the occurrence of the illusion. Based on the argument that depersonalization was a defense mechanism against danger and associated anxiety (Ananthaswamy, 2015/2018) and interpretation that individuals with depersonalization exhibited responses to suppress perception (including that of one’s own body) and cognition to maintain adaptive behavior in anxiety-related situations (Sierra and David, 2011), these results suggested that participants could have attempted to avoid perceiving the virtual body, presented with abdominal pain, as their own body, and thereby attenuated sensory input. Body illusions typically happen when the brain minimizes the mismatch (prediction error) between the predictions and actual sensory input by adjusting the prediction to match the sensory input (Apps and Tsakiris, 2014; Zeller et al., 2014, 2016; Rossi Sebastiano et al., 2021, 2024). However, the attenuation of sensory input by the negative top-down interpretation increased the prediction error between the top-down process “the virtual body in front of me is my body with abdominal pain” and sensory input. Consequently, the virtual body could not be modified as one’s own body, and the FBI may not have occurred.

Furthermore, we regard the possibility that the top-down interpretation of a mismatch between the state of the actual self-body and virtual body inhibited the occurrence of the illusion. Participants were asked to interpret the virtual body experiencing abdominal pain as their own body, despite their actual self-body not being in such a state. This discrepancy likely created cognitive conflict, which made it difficult for participants to accept the virtual body as their own. Difficulty in integrating the state of the virtual body with the state of their own body likely inhibited the FBI. From the perspective of predictive coding, this could be viewed as a mismatch between the predicted state (virtual body experiencing abdominal pain) and actual sensory input (actual self-body not experiencing abdominal pain). The body illusion arose as a result of correcting the prediction error between senses during the illusion procedure by assuming that the object was one’s own body (Apps and Tsakiris, 2014; Zeller et al., 2014, 2016; Rossi Sebastiano et al., 2021, 2024). However, owing to the manipulation of the top-down interpretation of the negative self, the above prediction error was also added. Furthermore, the brain struggled to resolve this error owing to the large discrepancy between the predicted state and actual state. Therefore, the brain attempted to correct the prediction error by not interpreting the virtual body as its own body. Consequently, the FBI may not have occurred.

We believe that our findings were consistent with those of previous studies that explained multisensory illusion phenomena via predictive encoding models. Our study extends this understanding by demonstrating that negative higher-order interpretations and depersonalizing features can gate this process and highlighting the important role of top-down influences in the formation of the sense of body ownership.

### 4.6 Limitations

This study had several limitations. First, although the “manipulation” of the negative top-down interpretation prevented FBI, it remains unclear whether the negative top-down interpretation or difference in the physical state between the current actual self-body and virtual body inhibits the creation of FBI. To examine whether the negative interpretation inhibits the creation of the FBI, future studies should establish the condition in which the virtual body is interpreted as a top-down self-body with negative physical symptoms and the condition in which the virtual body is interpreted as another physical state (e.g., a self-body with a positive state). Suppose that the degree of illusion is lower when the virtual body is interpreted as a negative self-physical state than when it is simply interpreted as a self-body and when it is interpreted as a positive self-physical state. In that case, the negative interpretation could be considered to have inhibited the illusion. However, suppose the degree of illusion is less when the virtual body is interpreted negatively or positively than when it is interpreted as the self-body. In that case, the difference between the state of the virtual body and actual self-body could be considered to have inhibited the illusion.

Second, how the fear stimuli used in this study influenced the top-down interpretation of the virtual body is unclear. Although this study did not confirm participants’ top-down interpretations of fear stimuli, presentation of fear stimuli may have led to a negative interpretation or interpretation of the difference between the state of the virtual body and actual self. Therefore, future studies should examine the type of top-down interpretation that the presentation of fear stimuli may have on a virtual body.

Third, there was a difference in the cognitive demands of the two tasks. Imagining abdominal pain was more cognitively demanding and required significant cognitive effort compared with the other condition. A trade-off exists between attention to internal thoughts and external stimuli, such as visual and tactile inputs (Ananthaswamy, 2015/2018). Considering this, the more demanding mental imagery of abdominal pain might have attenuated the visual-tactile input necessary for the occurrence of the illusion, or the visual input of the fear stimulus might have been attenuated, thereby inhibiting the occurrence of the illusion. Future studies should consider this cognitive load difference and examine its effects on the results.

Fourth, a more adequate sample size is required to clearly examine the relationship between depersonalization tendency and occurrence of the illusion. The relationship found in Yamamoto and Nakao’s (2022) study, which was also replicated in this study, was observed when the participants were divided into two groups, which indicated that the sample size was small. Therefore, to determine whether this can be replicated, an appropriate sample size should be calculated and examined without the influence of negative self-body interpretation on the neutral self-association condition. Furthermore, we acknowledge that our sample size was determined by previous research. After conducting a post-hoc power analysis, we found a power of 0.765. This indicated that the sample size may have been a limiting factor in fully confirming the hypothesized relationships. In addition, an adequate sample size would also lead to an appropriate interpretation of the relationship between depersonalization tendency and influence of negative self-body interpretations on the occurrence of illusions.

Finally, it is important to mention that the findings of this study were based on a third-person perspective FBI (Lenggenhager et al., 2007). Research has suggested that the FBI from a third-person view was considerably less effective than that from a first-person perspective (Petkova et al., 2011a, 2011b; Maselli and Slater, 2013, 2014; Gorisse et al., 2017). Unlike the first-person perspective FBI and rubber hand illusion, Lenggenhager’s third-person perspective FBI did not follow the basic spatial limitations of peri-personal space, which were crucial to body ownership illusions (Petkova et al., 2011b). Therefore, there may be fundamental differences between the third-person and first-person perspective FBI. Consequently, it is important to exercise caution when considering whether these findings can be applied to the first-person perspective FBI or RHI.

## 5 Conclusion

This study’s results are the first to indicate that a top-down interpretation inhibits the FBI. Beginning with Tsakiris and Haggard (2005), several studies have examined the influence of top-down interpretations on the RHI and FBI (Tsakiris et al., 2010; Guterstam et al., 2013; Yamamoto and Nakao, 2022). This study used a top-down interpretation with instructions that did not interfere with bottom-up input and found that even if the virtual body was manipulated to be interpreted as a self-body, the illusion was inhibited by the manipulation of the virtual body being interpreted as being in a negative physical state. However, it was unclear whether the negative top-down interpretation or difference in physical state between the self-body and virtual body inhibited the creation of the FBI. This study’s finding—specifically, that the FBI was inhibited by manipulating the top-down interpretation—suggested that top-down interpretation could be a factor inhibiting the creation of a sense of body ownership.

Additionally, this study suggested that the FBI, through top-down interpretation of the virtual body as a self-body, was associated with depersonalization tendencies. Combined with the finding that the FBI was inhibited when procedures were used for top-down interpretation of the virtual body as a self-body with negative physical symptoms, these findings suggested that negative self-body interpretation was related to the lack of sense of ownership of one’s body in depersonalization, based on a quantitative approach using the FBI. This suggests that the top-down interpretation of the self-body makes it difficult to experience a sense of body ownership and that an approach to improving top-down interpretation is key in depersonalization therapy.

## Data Availability

The raw data supporting the conclusions of this article will be made available by the authors, without undue reservation.

## References

[ref1] Althubaiti A. (2022). Sample size determination: a practical guide for health researchers. J. Gen. Fam. Med. 24, 72–78. doi: 10.1002/jgf2.600, PMID: 36909790 PMC10000262

[ref2] American Psychiatric Association (2013). Diagnostic and statistical manual of mental disorders. 5th Edn. Washington, DC: American Psychiatric Association.

[ref3] Ananthaswamy A. (2015/2018). The man who Wasn’t there: Investigations into the strange new science of the self. Translated by *R. Fujii* (Japan: Kinokuniya).

[ref4] Apps M. A. J. Tsakiris M. (2014). The free-energy self: a predictive coding account of self-recognition. Neurosci. Biobehav. Rev. 41, 85–97. doi: 10.1016/j.neubiorev.2013.01.029, PMID: 23416066 PMC3848896

[ref5] Benedek M. Kaernbach C. (2010). A continuous measure of phasic electrodermal activity. J. Neurosci. Methods 190, 80–91. doi: 10.1016/j.jneumeth.2010.04.028, PMID: 20451556 PMC2892750

[ref6] Berger C. C. Coppi S. Ehrsson H. H. (2023). Synchronous motor imagery and visual feedback of finger movement elicit the moving rubber hand illusion, at least in illusion-susceptible individuals. Exp. Brain Res. 241, 1021–1039. doi: 10.1007/s00221-023-06586-w, PMID: 36928694 PMC10081980

[ref7] Botvinick M. Cohen J. (1998). Rubber hands ‘feel’ touch that eyes see. Nature 391:756. doi: 10.1038/35784, PMID: 9486643

[ref8] Bresin K. Kling L. Verona E. (2018). The effect of acute physical pain on subsequent negative emotional affect: a meta-analysis. Personal. Disord. 9, 273–283. doi: 10.1037/per0000248, PMID: 28368146 PMC5624817

[ref9] Carey M. Crucianelli L. Preston C. Fotopoulou A. (2019). The effect of visual capture towards subjective embodiment within the full body illusion. Sci. Rep. 9:2889. doi: 10.1038/s41598-019-39168-4, PMID: 30814561 PMC6393432

[ref10] Ciaunica A. Seth A. Limanowski J. Hesp C. Friston K. J. (2022). I overthink—therefore I am not: an active inference account of altered sense of self and agency in depersonalisation disorder. Conscious. Cogn. 101:103320. doi: 10.1016/j.concog.2022.103320, PMID: 35490544 PMC9130736

[ref11] Costantini M. (2014). Body perception, awareness, and illusions. Wiley Interdiscip. Rev. Cogn. Sci. 5, 551–560. doi: 10.1002/wcs.1303 26308744

[ref12] de Jong J. R. Keizer A. Engel M. M. Dijkerman H. C. (2017). Does affective touch influence the virtual reality full body illusion? Exp. Brain Res. 235, 1781–1791. doi: 10.1007/s00221-017-4912-9, PMID: 28289799 PMC5435799

[ref13] Ehrsson H. H. Wiech K. Weiskopf N. Dolan R. J. Passingham R. E. (2007). Threatening a rubber hand that you feel is yours elicits a cortical anxiety response. Proc. Natl. Acad. Sci. USA 104, 9828–9833. doi: 10.1073/pnas.0610011104, PMID: 17517605 PMC1887585

[ref14] Finotti G. Garofalo S. Costantini M. Proffitt D. R. (2023). Temporal dynamics of the rubber hand illusion. Sci. Rep. 13:7526. doi: 10.1038/s41598-023-33747-2, PMID: 37160924 PMC10170134

[ref15] Gallagher S. (2000). Philosophical conceptions of the self: implications for cognitive science. Trends Cogn. Sci. 4, 14–21. doi: 10.1016/S1364-6613(99)01417-5, PMID: 10637618

[ref16] Gorisse G. Christmann O. Amato E. A. Richir S. (2017). First- and third-person perspectives in immersive virtual environments: presence and performance analysis of embodied users. Front. Robot. AI. 4:33. doi: 10.3389/frobt.2017.00033 PMC7805911 33501025

[ref17] Guterstam A. Björnsdotter M. Gentile G. Ehrsson H. H. (2015). Posterior cingulate cortex integrates the senses of self-location and body ownership. Curr. Biol. 25, 1416–1425. doi: 10.1016/j.cub.2015.03.059, PMID: 25936550

[ref18] Guterstam A. Gentile G. Ehrsson H. H. (2013). The invisible hand illusion: multisensory integration leads to the embodiment of a discrete volume of empty space. J. Cogn. Neurosci. 25, 1078–1099. doi: 10.1162/jocn_a_00393, PMID: 23574539

[ref19] Haans A. IJsselsteijn W. de Kort Y. (2008). The effect of similarities in skin texture and hand shape on perceived ownership of a fake limb. Body Image 5, 389–394. doi: 10.1016/j.bodyim.2008.04.003, PMID: 18650135

[ref20] Horn M. Fovet T. Vaiva G. Thomas P. Amad A. D'Hondt F. (2020). Emotional response in depersonalization: a systematic review of electrodermal activity studies. J. Affect. Disord. 276, 877–882. doi: 10.1016/j.jad.2020.07.064, PMID: 32739705

[ref21] Hunter E. C. M. Phillips M. L. Chalder T. Sierra M. David A. S. (2003). Depersonalisation disorder: a cognitive–behavioural conceptualisation. Behav. Res. Ther. 41, 1451–1467. doi: 10.1016/S0005-7967(03)00066-4, PMID: 14583413

[ref22] Kilteni K. Bergstrom I. Slater M. (2013). Drumming in immersive virtual reality: the body shapes the way we play. IEEE Trans. Vis. Comput. Graph. 19, 597–605. doi: 10.1109/TVCG.2013.29, PMID: 23428444

[ref23] Kilteni K. Maselli A. Kording K. P. Slater M. (2015). Over my fake body: body ownership illusions for studying the multisensory basis of own-body perception. Front. Hum. Neurosci. 9:119452. doi: 10.3389/fnhum.2015.0119452 PMC4371812 25852524

[ref24] Lakens D. (2022). Sample size justification. Collabra Psychol 8:33267. doi: 10.1525/collabra.33267

[ref25] Lenggenhager B. Tadi T. Metzinger T. Blanke O. (2007). Video ergo sum: manipulating bodily self-consciousness. Science 317, 1096–1099. doi: 10.1126/science.1143439, PMID: 17717189

[ref26] Maselli A. Slater M. (2013). The building blocks of the full body ownership illusion. Front. Hum. Neurosci. 7:83. doi: 10.3389/fnhum.2013.00083, PMID: 23519597 PMC3604638

[ref27] Maselli A. Slater M. (2014). Sliding perspectives: dissociating ownership from self-location during full body illusions in virtual reality. Front. Hum. Neurosci. 8:693. doi: 10.3389/fnhum.2014.00693, PMID: 25309383 PMC4161166

[ref28] Michal M. Koechel A. Canterino M. Adler J. Reiner I. Vossel G.. (2013). Depersonalization disorder: disconnection of cognitive evaluation from autonomic responses to emotional stimuli. PLoS One 8:e74331. doi: 10.1371/journal.pone.0074331, PMID: 24058547 PMC3772934

[ref29] Mottelson A. Muresan A. Hornbaek K. Makransky G. (2023). A systematic review and meta-analysis of the effectiveness of body ownership illusions in virtual reality. ACM Trans. Comput. Hum. Interact. 30, 1–42. doi: 10.1145/3590767, PMID: 39076787

[ref30] Nakul E. Orlando-Dessaints N. Lenggenhager B. Lopez C. (2020). Measuring perceived self-location in virtual reality. Sci. Rep. 10:6802. doi: 10.1038/s41598-020-63643-y, PMID: 32321976 PMC7176655

[ref31] Petkova V. I. Björnsdotter M. Gentile G. Jonsson T. Li T. Q. Ehrsson H. H. (2011a). From part- to whole-body ownership in the multisensory brain. Curr. Biol. 21, 1118–1122. doi: 10.1016/j.cub.2011.05.022, PMID: 21683596

[ref32] Petkova V. I. Ehrsson H. H. (2008). If I were you: perceptual illusion of body swapping. PLoS One 3:e3832. doi: 10.1371/journal.pone.0003832, PMID: 19050755 PMC2585011

[ref33] Petkova V. I. Khoshnevis M. Ehrsson H. H. (2011b). The perspective matters! Multisensory integration in ego-centric reference frames determines full-body ownership. Front. Psychol. 2:35. doi: 10.3389/fpsyg.2011.00035, PMID: 21687436 PMC3108400

[ref34] Quigley L. Warren J. T. Townsend C. (2022). Features of depersonalization: an examination and expansion of the cognitive-behavioral model. Psychol. Conscious. 11, 193–208. doi: 10.1037/cns0000336

[ref35] Roel Lesur M. Gaebler M. Bertrand P. Lenggenhager B. (2018). The plasticity of the bodily self: head movements in bodily illusions and their relation to Gallagher’s body image and body schema. Constr. Found. 14, 94–105.

[ref36] Romano D. Pfeiffer C. Maravita A. Blanke O. (2014). Illusory self-identification with an avatar reduces arousal responses to painful stimuli. Behav. Brain Res. 261, 275–281. doi: 10.1016/j.bbr.2013.12.049, PMID: 24412686

[ref37] Rossi Sebastiano A. R. Bruno V. Ronga I. Fossataro C. Galigani M. Neppi-Modona M.. (2021). Diametrical modulation of tactile and visual perceptual thresholds during the rubber hand illusion: a predictive coding account. Psychol. Res. 86, 1830–1846. doi: 10.1007/s00426-021-01608-0, PMID: 34773491

[ref38] Rossi Sebastiano A. R. Poles K. Gualtiero S. Romeo M. Galigani M. Bruno V.. (2024). Balancing the senses: electrophysiological responses reveal the interplay between somatosensory and visual processing during body-related multisensory conflict. J. Neurosci. 44:e1397232024. doi: 10.1523/JNEUROSCI.1397-23.2024, PMID: 38508711 PMC11079966

[ref39] Sierra M. (2009). Depersonalization: a new look at a neglected syndrome. Cambridge: Cambridge University Press.

[ref40] Sierra M. Berrios G. E. (2000). The Cambridge depersonalisation scale: a new instrument for the measurement of depersonalisation. Psychiatry Res. 93, 153–164. doi: 10.1016/S0165-1781(00)00100-1, PMID: 10725532

[ref41] Sierra M. David A. S. (2011). Depersonalization: a selective impairment of self-awareness. Conscious. Cogn. 20, 99–108. doi: 10.1016/j.concog.2010.10.018, PMID: 21087873

[ref42] Sugiura M. Hirosawa M. Tanaka S. Nishi Y. Yamada Y. Mizuno M. (2009). Reliability and validity of a Japanese version of the Cambridge depersonalization scale as a screening instrument for depersonalization disorder. Psychiatry Clin. Neurosci. 63, 314–321. doi: 10.1111/j.1440-1819.2009.01939.x, PMID: 19566762

[ref43] Tanabe H. (2004). Understanding pathological dissociation using the des scale (in Japanese). Jpn J. Clin. Psychiatr. 33, 293–307.

[ref44] Tsakiris M. Carpenter L. James D. Fotopoulou A. (2010). Hands only illusion: multisensory integration elicits sense of ownership for body parts but not for non-corporeal objects. Exp. Brain Res. 204, 343–352. doi: 10.1007/s00221-009-2039-3, PMID: 19820918

[ref45] Tsakiris M. Haggard P. (2005). The rubber hand illusion revisited: visuotactile integration and self-attribution. J. Exp. Psychol. Hum. Percept. Perform. 31, 80–91. doi: 10.1037/0096-1523.31.1.80, PMID: 15709864

[ref46] Yamamoto K. Nakao T. (2022). Top-down influence leads to a reduced sense of body ownership in individuals with depersonalization tendencies: a focus on full body illusion. Front. Psychol. 13:790960. doi: 10.3389/fpsyg.2022.790960, PMID: 35719590 PMC9201777

[ref47] Zeller D. Friston K. J. Classen J. (2016). Dynamic causal modeling of touch-evoked potentials in the rubber hand illusion. NeuroImage 138, 266–273. doi: 10.1016/j.neuroimage.2016.05.065, PMID: 27241481

[ref48] Zeller D. Reiners K. Bräuninger S. Buttmann M. (2014). Central motor conduction time may predict response to fampridine in patients with multiple sclerosis. J. Neurol. Neurosurg. Psychiatry 85, 707–709. doi: 10.1136/jnnp-2013-306860, PMID: 24357684 PMC4033025

